# Playing Around the Coumarin Core in the Discovery of Multimodal Compounds Directed at Alzheimer’s-Related Targets: A Recent Literature Overview

**DOI:** 10.3390/molecules30040891

**Published:** 2025-02-14

**Authors:** Mariagrazia Rullo, Gabriella La Spada, Angela Stefanachi, Eleonora Macchia, Leonardo Pisani, Francesco Leonetti

**Affiliations:** Department of Pharmacy—Pharmaceutical Sciences, University of Bari Aldo Moro, Via E. Orabona, 4, 70125 Bari, Italy; mariagrazia.rullo@uniba.it (M.R.); gabriella.laspada@uniba.it (G.L.S.); angela.stefanachi@uniba.it (A.S.); eleonora.macchia@uniba.it (E.M.); francesco.leonetti@uniba.it (F.L.)

**Keywords:** coumarin, multitarget ligands, Alzheimer’s disease, enzyme inhibition, metal chelators

## Abstract

Alzheimer’s disease (AD) causes a great socioeconomic burden because of its increasing prevalence and the lack of effective therapies. The multifactorial nature of AD prompts researchers to search for new strategies for discovering disease-modifying therapeutics. To this extent, the multitarget approach holds the potential of synergic or cooperative activities arising from compounds that are properly designed to address two or more pathogenetic mechanisms. As a privileged and nature-friendly scaffold, coumarin has successfully been enrolled as the heterocyclic core in the design of multipotent anti-Alzheimer’s agents. Herein, we comprehensively summarize the most recent literature (2018–2023), covering the rational design and the discovery of coumarin-containing multitarget directed ligands (MTDLs) whose anti-AD profile encompassed at least two different biological activities relevant for disease onset and progression. To enhance the clarity of presentation, synthetic coumarin-based MTDLs are categorized into four clusters based on their substitution pattern and reported bioactivities: (i) mono-, (ii) di-, and (iii) polysubstituted coumarins directed at protein targets, and (iv) coumarins directed at protein targets with additional metal-chelating features. Before discussing multimodal coumarins, the rationale for addressing each biological target is briefly presented.

## 1. Alzheimer’s Disease and Related Targets: Rationale

Alzheimer’s disease (AD) is a progressive and mostly irreversible disorder that impairs key functions within the central nervous system (CNS) as a consequence of peculiar shrinkage, affecting areas that control memory and learning. The discovery of new and effective drugs for curing AD represents one of the major challenges for researchers [1,2]. Initially, great emphasis was given to the cholinergic hypothesis [3] and the amyloid theory [4] to understand the several abnormalities featured by AD brains [5]. Unfortunately, the complex and multifactorial nature of the disease hampered the identification of novel drugs as real game changers. In the last two decades, the road towards drugs’ approval was plenty of failures and the research scored uncertain progresses [6]. Unsuccessful drug discovery prompted researchers to open new avenues toward the treatment of multifaceted neurodegenerative pathologies, such as combination approaches [7]. Within this strategy, an MTDL can be considered a polypharmacology-by-design molecular tool [8]. Indeed, the molecular framework of multitargeting ligands is rationally built to enable the simultaneous modulation of two (or more) targets relevant to the disease. Different proteins with enzymatic (e.g., cholinesterases), self-aggregating (beta-amyloid peptide), and receptor activity (e.g., histamine and adenosine receptors) have been considered viable targets for anti-Alzheimer’s drug candidates. Here, we only focused our attention toward biological targets for which coumarin-based MTDLs have been reported so far. AD-relevant bioactivities encompass target enzymes (e.g., cholinesterases, monoamine oxidases, carbonic anhydrases), receptors (e.g., cannabinoid receptors 1 and 2), aggregation-prone peptides (e.g., β-amyloid), and the chelation of redox metal species. Briefly, their rational use against AD is discussed.

### 1.1. Cholinergic System

For a long time, the cholinergic hypothesis has been the most accredited theory proposed to explain AD etiopathogenesis. It refers to a reduction in acetylcholine (ACh) neuronal levels, resulting in impaired cholinergic transmission. The catabolic degradation of ACh can be catalyzed by two serine-hydrolases, named acetyl- and butyrylcholinesterase (AChE and BChE). The main role of AChE is the termination of nerve impulse transmission at the synaptic level. The cholinergic system controls language, learning, and memory processes, and its regulation can be accomplished with neurotransmitter-sparing molecules [9], in particular AChE inhibitors. In addition, AChE promotes the formation and precipitation of beta-amyloid (Aβ) fibrils [10], and AChE-Aβ complexes demonstrated greater neurotoxicity than amyloid fibrils alone [11]. More recently, great attention has been directed to BChE, whose increased activity correlates with disease progression [12] and compensates AChE activity [13]. BChE is prevalently found in white matter and glial cells [14], is significantly recruited and activated in AD [15], and possesses amyloidogenic features [16]. High levels of BChE are associated with Aβ plaques and neurofibrillary tangles [16,17,18], which are typical AD hallmarks. Moreover, this isoenzyme can regulate the activation of the endocannabinoid system [19].

### 1.2. Amyloidogenic Pathways

The β-amyloid peptide (Aβ_40–42_) is composed of 40–42 amino acids and is produced by the cleavage of the amyloid precursor protein (APP), synthesized by blood cells, neurons, vascular cells, and astrocytes. Three different proteases (α-, β- and γ-secretase) can cleave the APP along two different pathways: 1) the non-amyloidogenic pathway, where nontoxic fragments (e.g., sAPP-α and C83) are produced by the sequential action of α- and γ-secretase; 2) the amyloidogenic pathway, where β-secretase (also known as BACE1 (β-site APP-cleaving enzyme 1)) forms sAPP-β peptides that are subsequently cleaved by γ-secretase, producing neurotoxic fragments. Among the latter, Aβ_42_ can aggregate into neurotoxic oligomeric fibrils ultimately resulting in senile plaques, one of the hallmarks of AD diagnosis [20].

### 1.3. Neurofibrillary Tangles

Tau is a microtubule-associated protein present in astrocytes, dendrites, and glia cells. It is responsible for the stabilization of microtubules and the maintenance of cytoskeletal architecture. In physiological conditions, tau binds to microtubules and enhances axonal transport, whereas in pathological conditions (termed tauopathies, e.g., Alzheimer’s disease) its increased phosphorylation determines its separation from microtubules and abnormal assembly into neurotoxic aggregates called neurofibrillary tangles (NFTs) [21]. Among the protein kinases enabling tau modification by phosphorylation, glycogen synthase kinase-3β (GSK-3β), a serine-threonine kinase found to be active in AD patients’ brain [22], has received a great deal of attention.

### 1.4. The Endocannabinoid System

Endocannabinoids (eCBs, e.g., anandamide) are lipid signalling molecules that regulate several physiological functions, e.g., neuroprotection, memory, cognition, pain, and the immune system. Recent lines of evidence suggest that the endocannabinoid system (eCS) is a key player in AD onset and progression. eCS comprises the endogenous cannabinoids (eCBs) and their canonical receptors (G-protein-coupled receptors, CB1 and CB2 receptors), the noncanonical receptors (TRPV, GPR18, GPR55, GPR119, PPARs), and the enzymes responsible for the synthesis and degradation of eCBs (fatty acid amide hydrolase, FAAH; monoacylglycerol lipase, MAGL) [23]. CB1 receptors are primarily found into the CNS, such as in the hippocampus and cortex, and their expression is not markedly influenced by AD. On the other side, CB2 receptors are mainly linked to immune cells (e.g., microglia), where they are upregulated during neurodegenerative and neuroinflammatory processes [24]. The release of anti-inflammatory mediators and the inhibition of pro-inflammatory cytokines is promoted by CB2R in both microglial cells and activated macrophages [25]. The stimulation of CB receptors, resulting in neuromodulation (via CB1R) and anti-inflammatory effects (via CB2R), can also be accomplished through FAAH blockade, thus maintaining high levels of anandamide and promoting neurogenesis. Moreover, excess amyloid peptide can induce FAAH overexpression [26].

### 1.5. Neuroinflammatory Mechanisms

Recently, increasing lines of evidence support the idea that a strong inflammatory component is present in AD pathogenesis [27]. High levels of pro-inflammatory mediators have been reported in the blood and cerebrospinal fluids of AD patients as the product of innate immune cells activity, mainly microglia. Starting from polyunsaturated fatty acids, the enzymatic activity of cyclooxygenases 1 and 2 (COX-1 and COX-2) and lipoxygenases (LOXs, in particular 5-LOX and 12/15-LOX) enables the formation of lipid signalling molecules (termed eicosanoids), which are associated with inflammatory processes. Moreover, LOXs are upregulated in Alzheimer’s disease and are correlated with amyloid payload and oxidative stress [28,29]. Similarly, in AD brains, the overexpression of COX-2 in the frontal cortex [30] promotes the amyloidogenic processing of APP [31]. Selective COX-1 inhibitors were found to counteract neuroinflammation in mouse models [32].

### 1.6. Oxidative Stress

Mitochondrial dysfunction and oxidative stress are deemed to be triggering factors rather than consequences of AD. To this extent, drugs that counteract reactive oxygen species (ROS) formation are regarded as viable disease-modifying tools. On this basis, the enzymatic activity of carbonic anhydrases (CAs) and monoamine oxidases (MAOs) deserves attention. CAs are iron-containing metalloenzymes that catalyze the reversible hydration of CO_2_ into bicarbonate ion, thereby regulating pH homeostasis. CA exists in different isoforms and is a well-known enzymatic target modulated by several coumarin-type inhibitors, in most cases irreversible. CA inhibitors are able to reduce cognitive decline by interfering with amyloid-related mitochondrial damage [33]. In particular, CA II is overexpressed in aged brain tissues and is found abundantly in post-mortem amyloid plaques [34]. CA VA and CA VB represent membrane-bound isoforms crucial for antioxidant defensive balance. CA VII is abundant within the CNS and is involved in neurodegeneration. Along with CA modulation, the inhibition of MAOs (basically, the central MAO B isoform) represents another strategy to restore the correct balance between harmful pro-oxidant and protective antioxidant species. MAOs are mitochondrial membrane-bound enzymes that contribute ROS precursors, thus promoting both neuronal death and neuroinflammation [35]. MAO B levels are elevated with ageing [36] and induce neuronal apoptosis. During AD progression, hippocampal and cortical regions show increased MAO B activity, reflecting cell loss and gliosis [37]. MAO B is also linked to amyloid deposition [38].

### 1.7. Bio-Metals Chelation

The correlation between AD and metal dyshomeostasis was corroborated by post-mortem analyses of amyloid plaques that revealed higher copper, iron, and zinc levels than healthy brain tissue [39]. Zinc and copper regulate Aβ production via distinct mechanisms influencing APP processing and accelerate its aggregation [40]. Metal ions can play a significant role in oxidative stress imbalance by sustaining ROS and reactive nitrogen species (RNS) production through Fenton and Haber–Weiss reactions. Cu^+2^ ions possess a high affinity for Aβ_1–42_ and accelerate senile plaques formation [41]. Additionally, metal ions are associated with neuroinflammation [42] and contribute tau protein hyperphosphorylation by activating protein kinases and inhibiting protein phosphatase 2A [43].

## 2. Coumarin

Coumarin (2*H*-1-benzopyran-2-one) and its derivatives are quite common in nature, particularly as products of secondary metabolism in higher plants [44]. As a nature-friendly and chemically tractable building block, coumarin has been incorporated into different drug structures (the antibiotic novobiocin and the anticoagulant warfarin, just to name a few). Apart from the well-known von Pechmann and Knoevenagel reactions, enabling the cyclization step of the fused pyrone ring, a plethora of experimental protocols have been developed that allow for easier access to derivatized coumarins [45]. Indeed, each C_sp2_-H bond can be exploited as a ramification point to introduce molecular diversity all around the heterocyclic core. Thus, coumarin is highly recurrent in bioactive molecules as a scaffolding element [46] or as a pharmacophore unit [47]. Thanks to its synthetic versatility, the coumarin backbone can be found in antiviral [48], neuroprotective [35], anti-inflammatory [49,50], anticancer agents [51,52], and in many other therapeutics. In this review, we focused attention on literature reports published in recent years that described coumarin-containing compounds with multitarget anti-Alzheimer’s biological activities.

## 3. Coumarin-Based Multitarget Ligands

### 3.1. Monosubstituted Multitarget Coumarins

**Figure 1:**
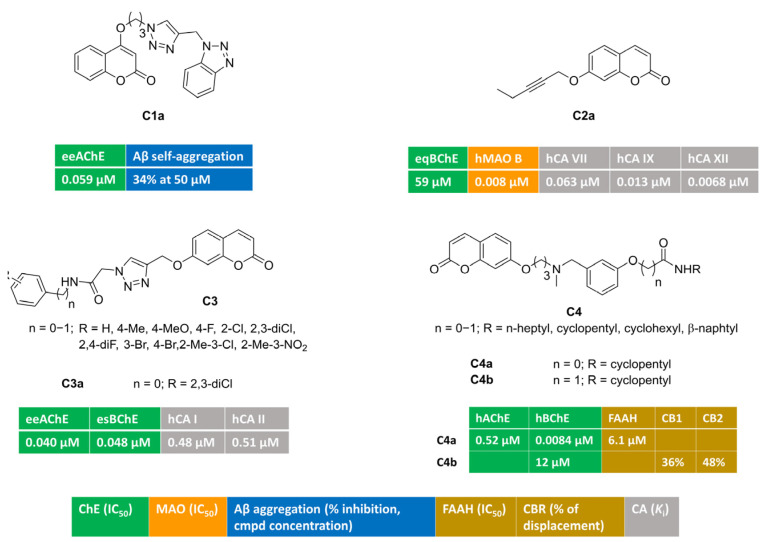
Monosubstituted multitarget coumarin derivatives **C1–4**.

Figure 1 illustrates multitargeting coumarins bearing different-sized monosubstitution patterns at position 4 (**C1**) or 7 (**C2–4**). A series of potent AChE inhibitors were discovered [53] by attaching a flexible triazole-containing polymethylene-linker at position 4 or 7 of the coumarin core to connect a terminal benzotriazole nucleus. The systematic investigation of linker chain length allowed for the identification of an oxypropyl tether as the optimal bridge, and the monosubstitution at position 4 yielded **C1a**. This compound was a potent AChE inhibitor (IC_50_ = 0.059 μΜ) endowed with bivalent metals chelation features and negligible activity toward BChE. At a high concentration, it was also able to reduce β-amyloid self- and copper-promoted oligomerization. Moreover, **C1a** exhibited mixed-type AChE inhibition kinetics. This was further supported by docking simulations, showing both peripheral and catalytic anionic subsite (PAS and CAS, respectively) occupancy.

Alkyl chains of different length (3–6 C-atoms), all containing an acetylene group, were attached at various positions (4-, 6-, 7-) of the coumarin ring [54]. With the aim of tackling neuroinflammation and ROS-related processes, Supuran and coll. tested these alkyl-coumarins for their capacity to block three biological targets (ChEs, MAOs, CAs). Particularly, MAO B inhibition was strongly affected by the branching position, with position 7 being the best choice. **C2a** behaved as a low nanomolar and selective MAO B inhibitor (IC_50_ = 0.008 μM), with poor activity toward BChE. Furthermore, it was quite active toward relevant CA isoforms, in particular, central CA VII and inflammatory CA IX and XII. It was devoid of activity toward cytosolic isoforms CA I and II. In silico prediction suggested that **C2a** could act as BBB-permeant molecule. It showed also a significant ability to decrease H_2_O_2_ production in rat astrocytes pre-stimulated with lipopolysaccharide (LPS), thus counteracting oxidative stress conditions.

By attaching a 1,2,3-triazoleacetamide moiety at coumarin position 7, the novel hybrids bearing general structure **C3** were obtained and evaluated as inhibitors of ChEs, carbonic anhydrase isoforms I and II, and other metabolic enzymes [55]. Notably, the blockade of AChE was at the nanomolar level. Derivative **C3a**, carrying a terminal hydrophobic 2,3-dichlorophenyl group, was a well-balanced nanomolar inhibitor of AChE (IC_50_ = 0.040 μM), BChE (IC_50_ = 0.048 μM), and cytosolic CA I (*K*_i_ = 0.48 μM) and II (*K*_i_ = 0.51 μM).

The modulation of eCS could be additive or synergistic with the inhibition of both ChEs resulting in a promising neuroprotection effect against cognitive diseases. Carbamate and amide groups were installed into **C4**, and multimodal coumarins **C4a**–**b** reported in [56] were found to be active against ChEs and eCS targets. Carbamate **C4a** was a potent BChE inhibitor (IC_50_ = 0.0084 μM), with additional inhibitory potency toward AChE and FAAH. Amide **C4b** showed a moderate BChE blockade and was able to modulate cannabinoid receptors (CB1 and CB2). The regulation of both CB1 and CB2 receptors might produce significant advantages in AD, being able to reduce amyloid toxicity via different mechanisms. In fact, CB1 stimulation reduces Aβ insults, whereas CB2 activation increases amyloid clearance and reduces microglia-related inflammation.

### 3.2. Disubstituted Multitarget Coumarins

**Figure 2:**
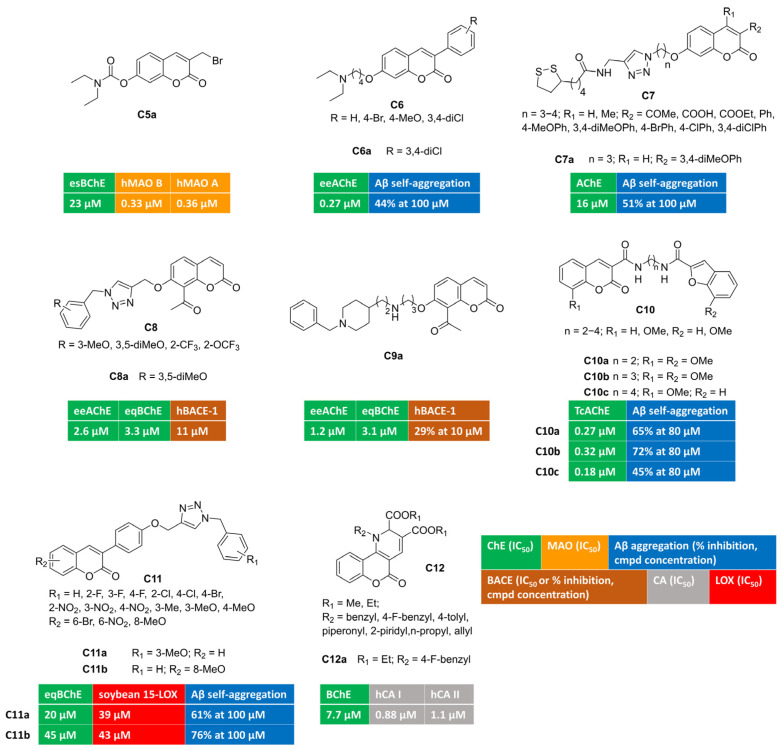
3,7-, 7,8-, 3,6-, 3,8-, 3,4-Disubstituted and 3,4-fused multitarget coumarin derivatives **C5–12**.

Aiming at developing dual covalent inhibitors of ChEs and MAOs [57], researchers introduced a carbamate moiety, inspired by pseudo-irreversible AChE inhibitors, at position 7, along with a propargylamine group introduced at position 3, to enable irreversible linkage with the FAD cofactor of MAOs. Unfortunately, these compounds were biased toward MAO B blockade, showing great B/A selectivity and negligible ChE inhibition. Then, a synthesis intermediate (bromide **C5a**, Figure 2), used as the starting material for propargylamine alkylation, was tested toward target enzymes. Surprisingly, **C5a** showed a multitargeting profile with moderate inhibition of BChE and nanomolar MAO inhibition without B/A selectivity. The mechanism of action of this electrophilic multimodal ligand was not investigated in the manuscript.

Different-sized aminoalkoxy chains were directly appended at position 7 (with 3-phenylsubstituted coumarin), or as the substituent branching the phenyl ring at position 3 of the coumarin ring [58] (Figure 2). 3,7-Disubstituted derivative **C6a**, bearing a 3,4-dichlorophenyl substituent at coumarin C3, was endowed with a submicromolar and selective AChE inhibitory potency (IC_50_ = 0.27 μM). It was able to span the AChE enzymatic cavity from CAS to PAS, as proved by the mixed-type inhibition found in the kinetics investigation. Additionally, it performed other anti-Alzheimer activities being able to reduce amyloid self-aggregation.

Researchers from Teheran University exploited azide–alkyne click chemistry to build a 1,2,3-triazole ring, connecting coumarin to lipoic acid, that could add antioxidant properties by means of metal-chelating and radical scavenging features. Different substituents were explored at coumarin C3 and C4 and the linker length was investigated (**C7**, Figure 2). Among novel coumarin–lipoic acid conjugates, **C7a** was the most potent, albeit moderate, AChE inhibitor. It displayed remarkable anti-amyloid properties, being able to block both self-induced and AChE-promoted Aβ oligomerization. Copper-chelating features for **C7a** were also proved by UV/Vis spectrometry. Neuroblastoma lines, insulted by hydrogen peroxide or Aβ_1–42_, were significantly protected when co-incubated with **C7a,** which increased cell viability. Moreover, it returned antioxidant activities comparable to those of well-known standard antioxidants quercetin and ascorbic acid in the ferric reducing antioxidant power (FRAP) assay and against intracellular ROS formation in PC12 cells stimulated with H_2_O_2_.

The plant kingdom represents the main source of coumarin derivatives as free forms or as glycosides. Naturally occurring 7-hydroxy-8-acetylcoumarin, a secondary metabolite isolated from *Nardostachys jatamansi*, was tested in vitro and showed the micromolar inhibition of AChE and BACE-1 activity [59]. Starting from this observation, cycloaddition reactions were used to functionalize 4-, 6-, and 7-OH groups, with a triazole linker anchoring diversely substituted phenyl rings [60] (**C8**, Figure 2). This approach led to improve in vitro enzymatic activities, yielding 7-substituted-8-acetylcoumarin/triazole hybrid **C8a** as a well-balanced multitargeting inhibitor (AChE, IC_50_ = 2.6 μM; BChE, IC_50_ = 3.3 μM; BACE-1, IC_50_ = 11 μM). Double-reciprocal plots of enzyme kinetics displayed non-competitive AChE inhibition and a mixed-mode blockade of both BChE and BACE-1 enzymatic activity. The self-aggregation of Aβ_1–42_ oligomers was slightly limited by **C8a**. A PAMPA (Parallel Artificial Membrane Permeability Assay) method was carried out to assess blood–brain barrier penetration for **C8a**, acting as a BBB-permeant molecule.

Semi-synthetic transformations of 7-hydroxy-8-acetylcoumarin were also undertaken by performing the Claisen–Schmidt condensation of acetyl group with different benzaldehydes. Resulting chalcones, exhibiting trans-double-bond geometries, were screened against enzymes relevant for Alzheimer’s pathology and showed moderate IC_50_s towards BACE-1 and poor inhibition towards ChEs [59]. Discouraged by negligible effects towards AChE and BChE, a different approach was envisaged by designing coumarin-donepezil hybrids. Thus, the *N*-benzylpiperidine moiety, inspired by donepezil, was tethered to 7-OH-coumarins by means of aminoalkoxy spacers. Using this strategy, **C9a** (Figure 2) was discovered as low micromolar inhibitor of AChE and BChE (AChE, IC_50_ = 1.2 μM; BChE, IC_50_ = 3.1 μM). It crossed the BBB in PAMPA experiments, reduced amyloid oligomerization, and inhibited BACE-1 activity (29% of inhibition at 10 micromolar concentration).

A different privileged-motif, namely benzofuran, was coupled with coumarin to obtain conjugates **C10a**–**c** [61] (Figure 2) as submicromolar inhibitors of AChE (0.18 μM < IC_50_ < 0.32 μM) with the potential of diminishing the neurotoxicity of amyloid plaques by interfering with amyloid fibrillization, as demonstrated with the thioflavin-T fluorescence method. The multimodal character of these derivatives was further studied in a radical scavenging assay employing 2,2-diphenyl-1-picrylhydrazyl (DPPH) free radical ions. The presence of methoxy group(s) improved the antioxidant capacity of this series of coumarin-benzofuran conjugates, and derivatives **C10a**–**c** were among the most potent antioxidants of the subset.

Looking at AD as an inflammatory pathology, a valuable target is represented by the inhibition of the prevalent central isoform of lipoxygenases (15-LOX), responsible for eicosanoid signalling along the arachidonic acid cascade. The simultaneous modulation of cholinergic transmission (through BChE inhibition), amyloid deposition, and inflammatory processes (through LOX inhibition) was achieved by 3-arylcoumarins bearing *N*-benzyl triazole moieties [62] (**C11**, Figure 2). Different substituents were arranged, either on benzyl group or on the coumarin ring, without promoting a remarkable activity improvement. Studied compounds were inactive toward AChE, and, in particular, **C11a**-**b** showed moderate dual-targeting inhibitory activity (BChE, IC_50_ = 20 and 45 μM, respectively; 15-LOX, IC_50_ = 39 and 43 μM, respectively) along with anti-amyloid aggregating effects. Moreover, antioxidant activities were observed in cell-based experiments conducted on BV-2 cells injured by Aβ_1–40_.

Alternatively, dihydropyridines were fused with coumarin at position 3,4 [63]. This strategy led to identifying compound **C12a,** displayed in Figure 2, as a moderate BChE inhibitor that was endowed with the additional inhibition of cytosolic CA I and II.

**Figure 3:**
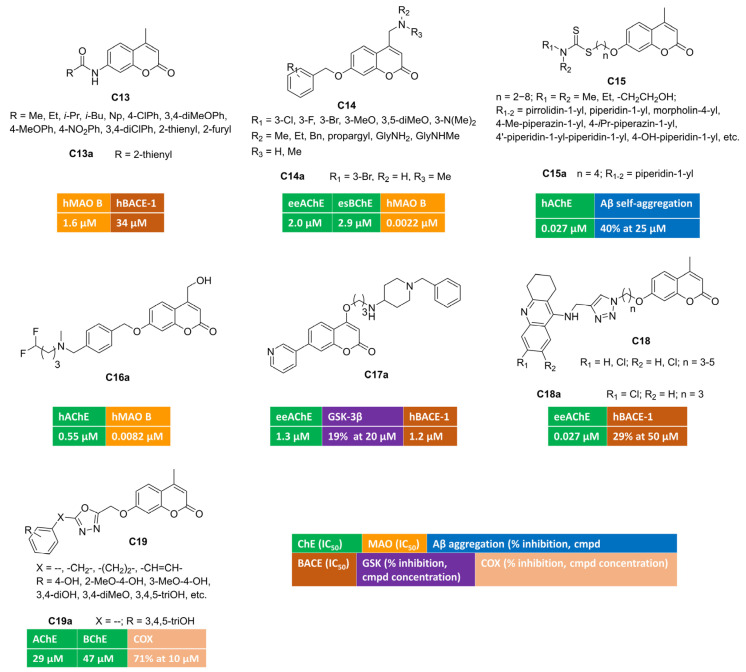
4,7-Disubstituted multitarget coumarin derivatives **C13–19**.

The acylation of 7-amino-4-methylcoumarin yielded different amides [64] that were screened against five enzymatic targets (AChE, BChE, MAO A and B, BACE) involved in AD pathology at different levels. Most of these novel compounds were inactive as ChEs and BACE inhibitors, whereas few single-targeting MAO B inhibitors were identified. In vitro enzymatic assays for compound **C13a** (Figure 3) displayed micromolar IC_50_s toward MAO B (IC_50_ = 1.6 μM) and BACE-1 (IC_50_ = 34 μM), thus corroborating its potential multimodal profile.

Alternatively, by introducing structural modifications, at position 4 (basic moiety) or 7 (benzyloxy tail) of a previously reported coumarin hit, some of us were able to discover triple-acting molecules [65], even if the bioactivity profile was unbalanced (**C14**, Figure 3). Indeed, for most of the benzyloxy congeners, the inhibitory potency toward one or both ChEs was in the micromolar range, whereas they were biased toward MAO B at the very low nanomolar level. One of the most active derivatives was represented by triple-acting coumarin **C14a** (AChE, IC_50_ = 2.0 μM; BChE, IC_50_ = 2.9 μM; MAO B, IC_50_ = 0.0022 μM).

A novel series of interesting AChE inhibitors was developed by linking 4-methyl-7-hydroxycoumarin with a dithiocarbamate group through a flexible polymethylene-chain [66]. In this work, compound **C15a** (Figure 3) emerged as potent AChE inhibitor with a mixed-type mechanism. Its multimodal profile included the ability to block the amyloid oligomerization at 25 micromolar concentration. Moreover, the sulphur-containing moiety promoted the biometal-chelation ability, in particular toward Fe^3+^ ions with an association constant equal to 1.69 × 10^3^ M^−1^. The stoichiometry of the complex was studied with the Job’s method, resulting in a 1:1 ratio. **C15a** proved to be BBB permeant in PAMPA experiments and safe in both cell-based models (human neuroblastoma lines) and in acute toxicity assays employing male Kunming mice (KM). When administered in vivo to KM, this hybrid was able to reverse the cognitive impairment induced by scopolamine in passive avoidance test.

The incorporation of fluorine and fluorinated motifs is a common practice in medicinal chemistry projects with the aim of modulating in vitro potency while controlling relevant ADME-properties (metabolic stability, solubility/lipophilicity balance, bioavailability) at the same time. Some of us investigated the impact of fluorine-based isosteres (H/F and CH_2_OH/CF_2_H) in a series of coumarin-based multitargeting inhibitors [67]. By applying isostere mimicry, we were able to identify a potent dual inhibitor (**C16a**, Figure 3) of AChE (IC_50_ = 0.55 μM) and MAO B (IC_50_ = 0.0082 μM), endowed with an outstanding MAO B/A selectivity. This compound bears a difluoromethyl group as a lipophilic hydrogen bonding donor and exhibits promising drug-like features encompassing high aqueous solubility, optimal lipophilicity, good metabolic stability, oral bioavailability, and favourable brain-permeation without P-gp efflux liability. Moreover, **C16a** was a non-cytotoxic agent able to counteract the oxidative damage triggered by different insults (hydrogen peroxide, NMDA, β-amyloid) over human neuroblastoma lines.

Multifunctional coumarin derivatives were designed by introducing differently substituted pyridines at position 7, whereas a flexible spacer connected a protonatable moiety, usually carrying a piperidine cycle, at position 4 of the alpha-pyrone ring [68]. Compound **C17a** (Figure 3) was almost 20-fold more active over AChE than BChE, with a low micromolar inhibitory potency and competitive inhibition kinetics, as indicated by Lineweaver–Burk plots. It displayed a moderate blockade of GSK-3β activity, which represents an important feature to delay upstream processes, leading to the formation of neurofibrillary tangles. In addition, **C17a** could interfere with the amyloidogenic processing of APP thanks to its promising activity toward BACE-1 (IC_50_ = 1.2 μM). This multimodal coumarin was moderately cytotoxic toward selected cell lines (HepG2, SH-SY5Y) and showed high BBB permeability. In vivo dose-scaling experiments unveiled low acute toxicity in mice for **C17a** that was tolerated up to 1g/kg dosage.

With the aim of obtaining AChE-targeting compounds, 4-methyl-7-hydroxycoumarin was linked to the tetrahydro-9-aminoacridine building block inspired by tacrine [69] (**C18**, Figure 3). The synthesis of these tacrine–coumarin hybrids employed azide–alkyne cycloaddition click chemistry to build the 1–4-disubstituted-1,2,3-triazole core within the flexible spacer, connecting the two heterocyclic scaffolds. As expected, the presence of tacrine moiety strongly influenced ChEs inhibition, and some nanomolar inhibitors of AChE or BChE were retrieved in this project. Compound **C18a** showed the highest AChE inhibition, with an IC_50_ value equal to 27 nM. It was also evaluated as BACE-1 inhibitor, resulting in a 29% inhibition at 50 μM concentration. Encouraged by these in vitro activities, **C18a** progressed toward animal models of amnesia induced by scopolamine. The results from Morris water maze test suggested that this hybrid could help in treating memory impairment, thus producing therapeutic benefits to people living with Alzheimer’s.

Another manuscript described polyhydroxylated or -methoxylated phenyl rings tethered to coumarin (position 3 or 7) by means of 1,3,4-oxadiazole-containing spacers [70] (general structure **C19**, Figure 3). Some of these polyphenolic derivatives were endowed with antioxidant properties. Among these, pyrogallol-based **C19a** moderately inhibited ChEs and was further tested against COX activity, returning an interesting 71% inhibition at 10 micromolar concentration.

### 3.3. Polysubstituted Multitarget Coumarins

**Figure 4:**
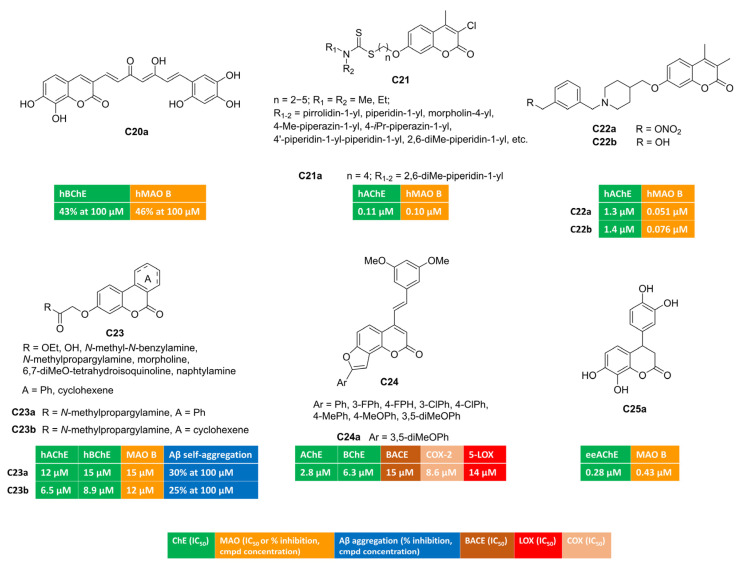
Polysubstituted multitarget coumarin derivatives **C20–25**.

Curcumin is a dietary polyphenol endowed with interesting antioxidant and anti-inflammatory properties. Moreover, its ability to disrupt or prevent protein aggregation, a worth-noting feature for anti-AD candidates, has been described. Its poor bioavailability represents the limiting step for its therapeutic usage. Researchers from the University of Santiago de Compostela designed a small series of curcumin–coumarin hybrids, which they synthesized and tested against ChEs and MAOs [71]. Most of the 3-(7-phenyl-3,5-dioxohepta-1,6-dien-1-yl)coumarin derivatives did not exhibit interesting multitarget profiles, as they behaved as single-targeting inhibitors of one the target enzymes. Free radical DPPH scavenging was also assessed. Only compound **C20a** illustrated in Figure 4 displayed comparable potency in inhibiting both BChE and MAO B isoform, showing 43% and 46% inhibition at 100 micromolar concentrations, respectively.

Coumarin–dithiocarbamate conjugates were exploited as multitarget agents for the treatment of Alzheimer’s disease [72]. Polymethylene chains of different length were investigated as flexible linkers connecting the coumarin scaffold with the dithiocarbamate moiety (**C21**, Figure 4). Most of these compounds demonstrated strong and selective inhibition towards AChE and MAO B. Notably, the four-carbon-atom linker provided compound **C21a** exhibiting well-balanced dual inhibitory activity against both AChE (IC_50_ = 0.11 µM, mixed-type inhibition) and MAO B (IC_50_ = 0.10 µM, reversible and competitive binding mode) along with good BBB penetration in PAMPA experiments and negligible toxicity in SH-SY5Y neuroblastoma cells. Moreover, **C21a** succeeded in reversing cognitive dysfunction in scopolamine-treated KM mice, returning a longer latency and fewer errors in the step-down passive avoidance test.

Some of us studied the potential of alkyl nitrates to work as precursors for alcohol-based dual AChE-MAO B inhibitors [67]. Non-enzymatic biotransformations promoted by active thiols (glutathione, cysteine residues) could unmask an alcohol (from a nitrate group) upon releasing NO, whose low doses can assist neuroprotective effects [73]. Three molecular fragments (coumarin, nitrate group, basic protonatable moiety) were combined and tested. Coumarin-based inhibitors bearing a different substitution at C7 (benzyloxy or 1-piperidin-3(4)-yl-methoxy groups) returned the best results. Remarkably, nitrate **C22a** (Figure 4) emerged as serum- and hydrolytically stable AChE-MAO B inhibitor, showing IC_50_ values equal to 0.051 μM and 1.3 μM toward MAO B and AChE, respectively. In the presence of glutathione, this derivative slowly released NO as well as the corresponding alcohol **C22b** (Figure 4), acting as dual inhibitor by itself (IC_50_ = 0.076 μM and 1.4 μM toward MAO B and AChE, respectively). Both alcohol and parent nitrate proved to be brain-permeant through passive diffusion. Bidirectional transport across MDCKII-MDR1 cells indicated CNS penetration without interaction with P-glycoprotein, as suggested by efflux ratio (< 2). Additionally, both **C22a** and **C22b** demonstrated protective effects when co-incubated with rotenone and hydrogen peroxide in human SH-SY5Y cultures, exhibiting low inherent cytotoxicity.

Gastric microflora can provide the catalytic machinery, enabling the biotransformation of polyphenolic substrates (i.e., ellagitannins from nuts, pomegranates) into OH-containing benzo[c]chromen-6-ones called urolithins. The functionalization of 3-hydroxy group yielded urolithin amides of general formula **C23** (Figure 4) that were tested toward ChEs and MAO B in search of novel multifunctional compounds as anti-AD hits [74]. Propargyl derivatives **C23a**–**b** displayed a well-balanced multitarget profile and were found active toward ChEs (AChE and BChE) and MAO B, albeit at the micromolar level. At high concentrations, they were able to disrupt the formation of amyloid fibrils, which represents a key feature in the treatment of Alzheimer’s disease. The benzene cycle fused to coumarin position 3 and 4 (**C23a**) was not essential for the enzymatic activities, and saturated analogue **C23b** returned similar IC_50_ values. Both amides were also antioxidants, performing radical chain breaking effect in oxygen radical absorbance capacity (ORAC) test.

COX blockade can be an alternative strategy to disrupt inflammatory cascade originating from cellular PUFA metabolism. A 3,5-dimethoxystylbene moiety was appended to differently substituted angular furocoumarins [75] and the resulting hybrids were tested as multifunctional anti-Alzheimer compounds, targeting ChEs, β-secretase, COX-2, and 5-LOX (**C24**, Figure 4). Derivative **C24a** proved to be a low micromolar inhibitor of different enzymes (AChE, BChE, BACE, COX-2, 5-LOX) and displayed additional scavenging ability against free radical species. This latter effect was observed in the DPPH assay and then confirmed in cell-based tests involving lipopolysaccharide (LPS)-induced oxidative stress in both MCF-7 and Hek293 cells.

In another work, the coumarin ring was decorated at C4 with diverse aryl motifs, returning dual ChE/MAO B inhibitors [76]. Surprisingly, 3,4-dihydrocoumarin **C25a** (Figure 4) emerged as a balanced dual AChE/MAO B inhibitor (AChE, IC_50_ = 0.28 μM; MAO B, IC_50_ = 0.43 μM), with antioxidant capacity in the FRAP assay, comparable to vitamin C and attributable to the polyhydroxylated skeleton.

### 3.4. Metal-Chelating Multitarget Coumarins

**Figure 5:**
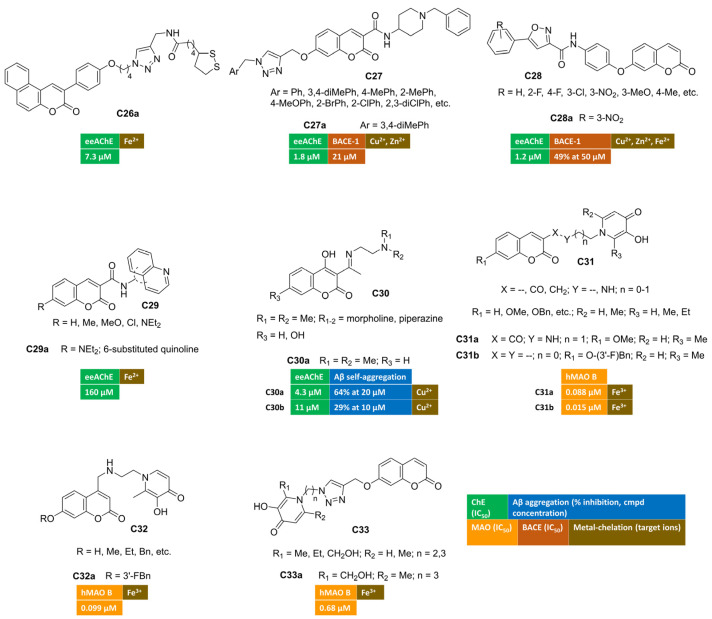
Metal-chelating multitarget coumarin derivatives **C26–33**.

A series of 3-arylcoumarin/lipoic acid conjugates was developed in search of multimodal agents for the treatment of AD. The spacer connecting the two heterocyclic cores was built through an azide–alkyne cycloaddition reaction, allowing the insertion of a triazole moiety to enrich the anti-Alzheimer’s activity with metal-chelating features [77]. The four-carbon spacer enabled AChE inhibition at the low micromolar range. Cell-based studies showed that many of the conjugates were able to protect PC12 cells from oxidative stress induced by H_2_O_2_. Benzo[f]coumarin **C26a** (Figure 5) emerged as the most potent AChE inhibitor within this series, and it was further endowed with an interesting antioxidant profile revealed by FRAP assay and 1:1 Fe^2+^-complex formation. Moreover, **C26a** showed protective effects against A*β*_1–42_-induced cell damage in SH-SY5Y lines.

Along a different molecular design strategy, a triazole-containing motif was introduced at the position 7 of coumarin-3-carboxamides, bearing a donepezil-inspired basic tail [78]. Among hybrids with general structure **C27** (Figure 5), compound **C27a** displayed the highest AChE inhibitory potency (IC_50_ = 1.80 µM) and provided a mixed-type inhibition behaviour in the kinetic study. Its moderate activity towards BACE-1 (IC_50_ = 21 μM) claims for an additional anti-AD activity, that is the disruption of the amyloidogenic processing of APP ultimately leading to oligomers’ fibrillization and deposition. Thanks to the triazole-group, UV/Vis spectra of **C27a** were characterized by a hypsochromic effect (blue shift) mainly in the presence of both Cu^2+^ and Zn^2+^ ions, unveiling the ability to chelate these metal species.

7-OH-Coumarin was linked to arylisoxazole-carboxamides, affording a novel class of ChEs inhibitors possessing general structure **C28** (Figure 5) [79]. Ellman’s method indicated that hybrid **C28a** was the most active AChE inhibitor (IC_50_ = 1.2 μM) with a competitive mode of inhibition, as can be inferred from the Lineweaver–Burk plot. Furthermore, the activity toward BACE-1 was evaluated in a FRET-based assay protocol, returning the 49% of inhibition at 50 micromolar concentration. Moreover, the authors reported changes in UV absorption spectra for this coumarin in the presence of bivalent ions, suggesting the complexation of studied redox metals (Zn^2+^, Fe^2+^, Cu^2+^).

A series of different hybrids were synthetized by amidation reactions, coupling coumarin-3-carboxilic acids with different aminoquinolines, thus yielding ChEs inhibitors active in the high micromolar range [80]. Additionally, some of the studied compounds exhibited promising iron-chelating properties, suggesting a potential neuroprotective effect. Compound **C29a** (Figure 5) proved to be an excellent iron-chelating agent.

The introduction of Schiff bases at C3 of 4-hydroxycoumarins **C30** (Figure 5) led to multifunctional anti-AD derivatives, such as **C30a**–**b** [81,82]. These compounds showed high selectivity towards AChE-inhibition (IC_50_ = 4.3 μM and 11 μM for **C30a** and **C30b**, respectively). They were also able to inhibit both the self-induced aggregation of A*β*_1–42_ (64% at 20 μM and 29% al 10 μM, respectively) and copper-promoted oligomerization. Notably, both imines had good metal chelating ability, showing an hypochromic shift in particular when Cu^2+^ ions were added to the ethanol of solutions of **C30a** [81]. In addition, the presence of hydroxyl groups enhanced their radical scavenging activity.

Other series of interesting conjugates were obtained from the combination of coumarin (as MAO B-inhibiting motif) with 3-hydroxypyridin-4-one ring as the metal-chelating fragment [83,84] (**C31** in Figure 5). Indeed, the incorporation of the nitrogen-containing ring through an amide- (**C31a**) or a methylene-linker (**C31b**) provided nanomolar MAO B inhibition (IC_50_ = 0.088 μM and 0.015 μM for **C31a** and **C31b**, respectively) with outstanding iron-chelating properties. Compound **C31a** displayed the most remarkable metal-chelating activity with pFe^3+^ value equal to 18.93, even higher than standard deferiprone (pFe^3+^ = 17.50). Moreover, this coumarin exhibited strong antioxidant activity, protected PC12 cells from oxidative damage induced by A*β*_1–42,_ and significantly ameliorated the cognitive decline induced by scopolamine in Morris water maze test. In a study by C. Zhang et al. [84], **C31b** returned cytoprotective effects against oxidative stress triggered by H_2_O_2_ in U251 cells and notably improved memory dysfunction in scopolamine-treated mice as animal model of AD.

By placing 3-hydroxypyridin-4(1*H*)-one pharmacophore at coumarin C4, compounds of general structure **C32** (Figure 5) were designed and synthesized [85]. In vitro assays indicated that all derivatives were endowed with good anti-MAO B activity and outstanding iron-chelating properties. The hit compound of this series (**C32a**) showed the best MAO B inhibitory potency (IC_50_ = 0.099 μM) along with iron-chelating features (pFe^3+^ = 17.1). It was also capable of increasing the cell survival rate (PC12 cell line) at 10 μM concentration after A*β*_1–42-_induced cellular damage and exerting antioxidant activity. Thanks to its promising multimodal profile, **C32a** was advanced toward behavioural studies enrolling scopolamine-treated mice where it enabled a significant memory enhancement. Moreover, promising pharmacokinetic parameters were recorded by means of UHPLC-MS/MS method after intravenous injection in rats.

Alternatively, hydroxypyridinone and coumarin pharmacophore were merged into the compounds bearing general structure **C33** (Figure 5) [86], where an additional chelating moiety (i.e., 1,2,3-triazole) was integrated into the linker through azide–alkyne click chemistry. The same bifunctional chelating moiety was also appended to positions 3 and 4 of the coumarin nucleus. The effects of the pyridinone ring’s substituent and the optimal distance with the coumarin were studied by measuring MAO B inhibitory activities and iron-chelating properties. Among these hybrids, **C33a** exhibited excellent iron-chelating activity (pFe^3+^ = 19.8). Furthermore, in vitro biological evaluation toward MAO B isoenzyme indicated a potent submicromolar inhibition, being IC_50_ = 0.68 µM.

## 4. Conclusions and Perspectives

Alzheimer’s disease treatment represents an open issue in current medicine. Its complex multifactorial etiology laid the groundwork for enrolling novel drug targets and innovative drug design approaches, such as MTDL strategy. Huge efforts have been dedicated to this research area by decorating the coumarin backbone with diverse substitution patterns to target multiple enzymatic pathways critical to the onset and/or progression of AD. The right choice of networked targets represents a bottleneck in designing effective MTDLs. This is much more complicated for anti-Alzheimer’s drugs, because disease comprehension is still elusive as proved by the lack of market approvals in the last two decades, except for monoclonal antibodies promoting amyloid clearance. However, most of designed hybrid molecules reported in this review have been directed to a restricted group of enzymes or mechanisms, giving much more attention to ChEs, MAOs, and amyloid aggregation inhibition compared to other biological targets. Whether this is the consequence of the appeal of old-fashioned targets and the easy access to well-consolidated assay protocols (kits and materials), rather than intended design approaches, is a matter of debate. Obviously, deeper efforts should also be committed to novel viable and druggable targets based on recent advances in the study of AD pathogenesis.

Herein, more recent literature reports (2018–2023) were comprehensively reviewed, highlighting the scaffolding properties of coumarin and the easy chemical tractability that allows the synthesis of multimodal anti-Alzheimer’s small molecules. Coumarin derivatives without precise target engagement, such as non-specific antioxidants or neuroprotectants, were not included in this manuscript.

The balance of multiple activities remains a great challenge in the field of MDTL discovery. In some cases, multitarget coumarin derivatives may be considered dirty or promiscuous drugs lacking balanced bioactivity profiles. In many other cases, the biological activities were at the same order of magnitude and deserve further attention.

Furthermore, great attention should be paid toward drug-like properties in order to enhance the success rate of drug discovery programmes. In many cases, conjugate and hybrid multitargeting coumarins can suffer from molecular obesity (high lipophilicity, high molecular size) that could hamper their further development. The assessment of ADME-properties is needed at early stage of projects, also taking toxicophore alerts into consideration. Even if the molecular design step can be aided by rational strategies (e.g., fragment- or structure-based design) to reduce molecular complexity or by in silico tools pointing out sub-optimal drug-likeness preliminarily, the employment of these approaches is clearly underestimated in the field. Noteworthy, we have reported some prototypes of multipotent coumarins showing drug-like pharmacokinetic features and promising outcomes in animal studies, giving new hope for AD therapy.
